# Fabrication and Characterization of Brain Tissue Phantoms Using Agarose Gels for Ultraviolet Vision Systems

**DOI:** 10.3390/gels10080540

**Published:** 2024-08-20

**Authors:** Luis M. Vidal-Flores, Miguel Reyes-Alberto, Efraín Albor-Ramírez, César F. Domínguez-Velasco, Enoch Gutierrez-Herrera, Miguel A. Padilla-Castañeda

**Affiliations:** 1 Applied Sciences and Technology Institute ICAT, National Autonomous University of Mexico UNAM, Ciudad Universitaria, Mexico City 04510, Mexico; luis.vidal@icat.unam.mx (L.M.V.-F.); efrain.albor@icat.unam.mx (E.A.-R.); cesar.dominguez@icat.unam.mx (C.F.D.-V.); 2 ABB Inc., 3400 Rue Pierre-Ardouin, Quebec, QC G1P 0B2, Canada; enoch.gutierrez-herrera@ca.abb.com

**Keywords:** UV vision, phantom, brain tissue-like phantom, UV, agarose

## Abstract

Recreating cerebral tissue using a tissue-mimicking phantom is valuable because it provides a tool for studying physiological and biological processes related to tissues without the necessity of performing the study directly in the tissue or even in a patient. The reproduction of the optical properties allows investigation in areas such as imaging, optics, and ultrasound, among others. This paper presents a methodology for manufacturing agarose-based phantoms that mimic the optical characteristics of brain tissue using scattering and absorbing agents and proposes combinations of these agents to recreate the healthy brain tissue optical coefficients within the wavelength range of 350 to 500 nm. The results of the characterization of the manufactured phantoms propose ideal combinations of the used materials for their use in controlled environment experiments in the UV range, following a cost-effective methodology.

## 1. Introduction

Imitating human tissue by producing phantoms with similar optical characteristics is a valuable tool for research focused on studying the interaction of light with different types of human tissues [1,2,3]. These topics could be carried out without the necessity of having real tissue or even a patient, using a phantom instead. This shows the need to develop phantoms with optical parameters as precisely as possible compared to real tissues.

The use of phantoms in the ultraviolet (UV) spectrum can facilitate a detailed study of this range of wavelengths, which opens the possibility of a deeper understanding of the physiology, pathologies, and biological processes in tissues [1,2,4]. When performing studies in a controlled environment, some materials allow us to better observe and analyze the optical behavior of tissue-mimicking samples. This encourages research in biology and medicine, which opens the way for the development of new studies, such as the detection of anatomical structures of interest and pathological tissues using spectroscopic techniques.

The study of biological tissues in the UV range has been demonstrated to be helpful because it can be used to identify some pathologies by detecting nicotinamide adenine dinucleotide (NADH) [5], for example, skin cancer [6] or some other diseases [7]. This technique helps in better resection of the pathological tissue. Some cellular and molecular changes can be detected before they are visible to the naked eye using medical imaging techniques [8,9,10]; however, light absorption is an important limitation in the UV spectrum of biological tissues [4,5,6,7,8,9,10,11] due to the properties of blood, which depend on components like hemoglobin and other pigments. Hemoglobin limits the penetration of light, reducing the information that can be obtained from biological structures [12,13].

In neurological surgery, procedures are performed on the brain with the cranial cephalic matter exposed, which reduces the amount of blood covering the tissue of interest and motivates the development of methodologies that recreate brain tissue using controlled parameters. The use of this phantom could help obtain high-quality images similar to biological tissues, allowing the validation and adjustment of systems before their clinical application and improving their precision and sensibility in the simulation of the specific conditions of a tissue, which may guarantee appropriate measures in clinical environments.

The use of fluorescence-guided surgery to aid in the identification of pathological tissues has gained attention in neurosurgery [14]. Fluorescence has been especially useful [8,9,10] because tumor pathologies are surrounded by healthy functional tissue [15], which often do not present changes in appearance. Therefore, a well-delimited tumor is required during medical intervention. Moreover, the autofluorescence technique is becoming a focus of research [9,16,17,18] because it is based on the detection of endogenous biomarkers, taking advantage of the autofluorescence properties of tissues in the ranges of near-ultraviolet excitation/emission. However, it is still in its early stages in neurosurgery; therefore, controlled and safe experimental environments would be valuable for future research in this field. Manufacturing tissue phantoms mimicking the brain’s optical characteristics will allow controlled experimentation environments.

Previous works report the optical characterization of brain tissue in the UV range. However, most of these are limited to 400 nm [2,19,20,21]. Honda et al. [22] tested four samples of normal human brain tissue, Yaroslavsky et al. [23] prepared 14 samples from 7 non-diseased human brains during autopsy, Li et al. [24] measured 55 glioma or normal brain tissue specimens from 19 patients, Gebhart et al. [25], 19 white matter samples and 25 gray matter samples, and Shapey et al. [26] tested nine different samples from a single subject undergoing a post-mortem examination.

In addition, works focused on developing phantoms of brain tissue are typically concentrated on reporting chemical, mechanical, and acoustic properties, and those reported with optical properties show values in wavelengths larger than 400 nm. Therefore, developing methodologies that help emulate brain tissue’s optical properties in the UV spectrum is essential for many clinical applications, such as those described above. This will be useful mainly for image acquisition systems focused on challenging-to-access tissues, such as tumors or cerebral tissue [27,28].

This will allow us to conduct better studies focused on brain tissue in the same wavelength range in a controlled environment, which could be used for calibration and training.

Different materials are used to manufacture synthetic phantoms. Polymers and those made of biological materials are the most common. Examples of polymers include polydimethylsiloxane (PDMS) [29] and silicone polyacrylamide gel (PAA) [30].

Biological materials such as gelatin or agarose are typically used to recreate biological tissues, given their similar properties like flexibility, deformability, and chemical and structural properties. These indicate that they are appropriate materials for creating similar mimicking tissues for a variety of uses. Furthermore, they have a similar response to mechanical stimuli. Some works show that the use of different types of materials can be useful for recreating specific characteristics by varying the material concentrations and utilizing them for various medical applications [31,32,33]. Applications range from the fabrication of tissue-mimicking phantoms for photoacoustic microscopy systems [31,32,33] to photodynamic therapy [34,35] or even as hydrogels for bioprinting [36].

Phantoms developed with biological materials are aimed at applications such as image acquisition, ultrasound, and dosimetry. Their use arises from their high water content, which favors their compatibility with other organic elements, such as NADH [37] or tryptophan [38], among other fluorophores or optical markers, widening their utility in many medical areas [39].

Within biological materials, agarose has been widely used to manufacture soft tissue phantoms because its mechanical properties can be adjusted by varying its concentration in terms of the volume of water used. For brain tissue phantoms, concentrations within the range of 0.6% to 1% *w*/*v* have been reported to resemble the mechanical properties of natural brain tissue [40,41].

The optical parameters that must be replicated when recreating human tissue include the absorption, ${µ}_{a}\left(\lambda \right)$, and reduced scattering, ${µ}_{s}^{\prime }\left(\lambda \right),$ coefficients. These parameters depend on the material used for manufacturing the phantom and the agents selected for tuning dispersion and absorption. There are limited works that tested brain tissue at wavelengths near or lower than 400 nm, which complicates the comparison of the phantom manufactured with the real tissue values.

The manufacturing and characterization of tissue-mimicking phantoms used for UV spectra have rarely been addressed. Most of the efforts have been focused on wavelengths larger than this range. With this motivation, this work proposes a methodology for manufacturing phantoms that imitate cerebral cortex tissue’s optical properties for its use within the UV range wavelengths. Agarose was selected as the phantom matrix in combination with Indian ink and titanium dioxide (TiO_2_) to fabricate synthetic tissue samples. The absorption and reduced scattering coefficients of such phantoms were characterized within the spectrum between 350 nm and 500 nm.

Our results indicate that it is possible to fabricate brain tissue phantoms with optical properties similar to those reported in the literature within the UV range of 350 to 500 nm. This opens the door to the design, development, and construction of multispectral vision systems for brain tissue monitoring, for example, for the detection of pathologies whose spectral response is within the range of interest, such as in fluorescence imaging.

The remainder of this paper is organized as follows. In Section 2, we present the results of the optical characterization. Section 2 presents a brief discussion of the findings. Section 3, the conclusion. Finally, in Section 4, we describe the fabrication and characterization methodology.

## 2. Results and Discussion

From the transmission and reflection tests, the absorption, ${µ}_{a}\left(\lambda \right)$, and reduced scattering, ${µ}_{s}^{\prime }\left(\lambda \right)$, coefficients were calculated to be used as the parameters to compare with brain tissue to determine the similarity with the biological tissue.

Particles of TiO_2_ were used as the scattering agent because of their wide use in fabricating tissue-mimicking phantoms. In addition, Indian ink was used as an absorption agent due to its widespread usage in this type of phantom, even other elements made from carbon.

### 2.1. Transmission and Reflection Tests with Agarose Films

To determine the light interaction characteristics of the phantom support matrix, the first tests that were carried out only used agarose gel films to characterize the basal behavior of the absorption or scattering of this gel as a matrix.

Therefore, five films were tested by transmission and reflection and plotted, expecting almost the complete percentage of the reference light in transmission and a meager rate in reflection, implying shallow values of the optical properties for this material.

**Figure 1:**
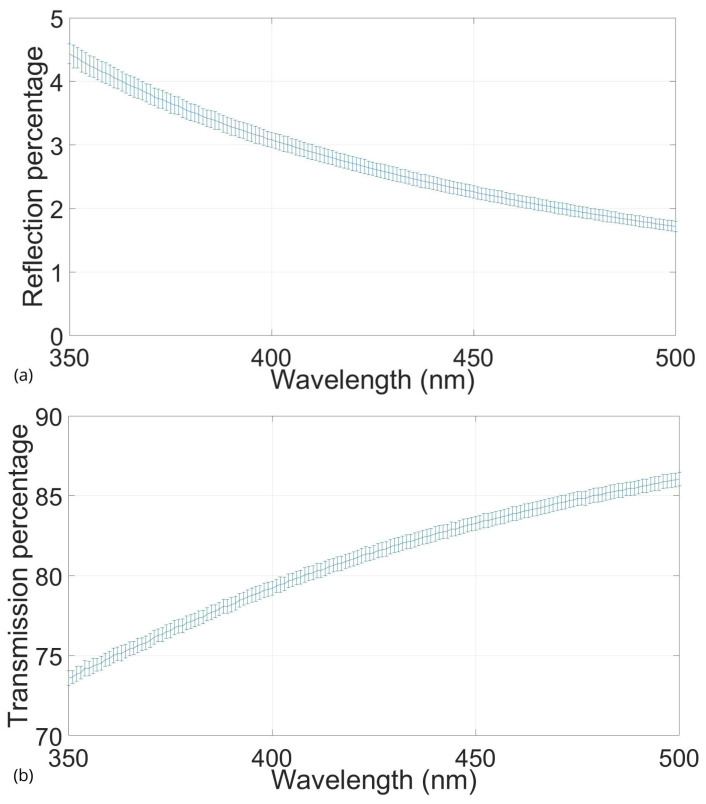
Average reflection percentage of five 0.7% (*w*/*v*) films of agarose (**a**). Average transmission percentage of five 0.7% (*w*/*v*) films of agarose (**b**).

Figure 1 shows a plot of the percentage of reference light transmitted through the samples and the percentage reflected from them. The spectrum between 350 nm and 500 nm was selected because, within it, most of the endogenous tissue fluorophores can be found [42,43,44,45,46], which is of interest for applying these phantoms in the development, experimental evaluation, and characterization of fluorescence imaging systems for brain monitoring and neurological applications.

The reflection shown in Figure 1 is nearly zero and thus vanishes in most of the observed range, as expected. In the same figure, the transmission decays by only 13% within the studied range, which indicates a high transmittance and low reflection from the agarose gel. The prior indicates that its contribution to the absorption and scattering coefficients is very small, which makes it a suitable material for use as a support matrix for recreating tissue-like phantoms, and its optical coefficients will depend only on the scattering and absorbing agents mixed with it.

In several works [40,41,47,48], concentrations in the range of 0.6% to 1% have been used to obtain mechanical properties that mimic the rigidity of brain tissue. Thus, in this work, our tissue samples were fabricated at concentrations of 0.7% agarose, considering that the concentration of 0.6% is the typical average concentration mostly reported in the literature. In our case, the decision to use a slightly higher concentration was based on empirical observations of the elastic behavior of the gel in our experimental configuration, from which we observed that by increasing its mechanical rigidity at a higher concentration, the samples were able to adhere stably to the cartridge that contained them better than with 0.6% samples. This avoids the risk of the samples becoming detached from the sphere used during their optical characterization, damaging the spectroscopy equipment.

Additionally, the motivation to maintain single concentrations of 0.7% agarose over the experiments was to guarantee the same mechanical stability in all samples but to concentrate on characterizing their optical properties.

### 2.2. Transmission and Reflection Tests with Agents

The same test shown previously must be performed with the films combined with the absorbing and scattering agents to verify the homogeneity of the samples of the different concentrations. In other words, testing the fabrication repeatability.

**Figure 2:**
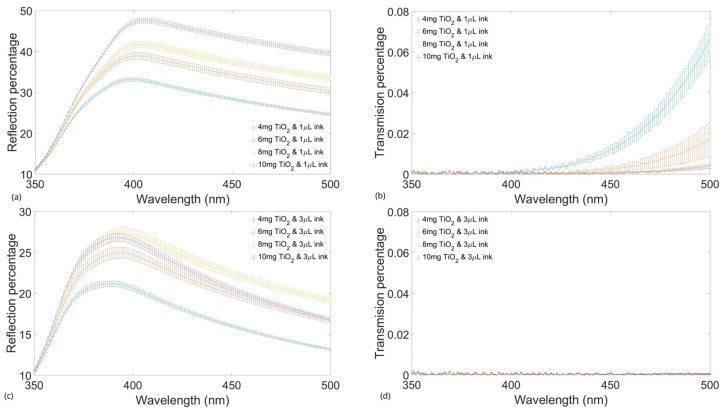
Reflection percentage of 0.7% (*w*/*v*) film of agarose at different concentrations of TiO_2_ and 1 µL of Indian ink (**a**). Transmission percentage of 0.7% (*w*/*v*) film of agarose at different concentrations of TiO_2_ and 1 µL of Indian ink (**b**). Reflection percentage of 0.7% (*w*/*v*) film of agarose at different concentrations of TiO_2_ and 3 µL of Indian ink (**c**). Transmission percentage of 0.7% (*w*/*v*) film of agarose at different concentrations of TiO_2_ and 3 µL of Indian ink (**d**).

Figure 2 shows the plots of the transmission and reflection percentage of the reference light of the films with different amounts of TiO_2_ and two different concentrations of ink. The graphs present the mean and standard deviation of the samples, showing the reliability of the fabrication process.

### 2.3. Absorbing and Scattering Agents Effects

**Figure 3:**
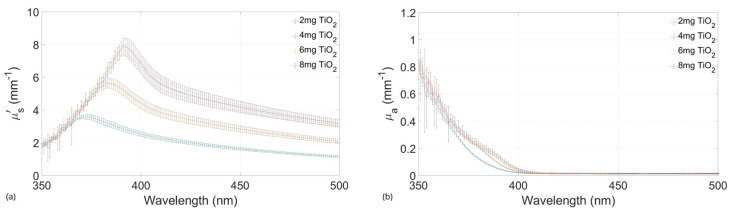
Absorption coefficients of agarose films at four different concentrations of TiO_2_. (**a**) Scattering coefficients of agarose at four different concentrations of TiO_2_. (**b**) Absorption coefficients of agarose films at four different concentrations of TiO_2_.

Developing a tissue-mimicking phantom requires absorption and scattering properties to be similar to those of the desired tissue. Therefore, the characterization of the samples with the agents was first performed only with TiO_2_, testing gels with different concentrations of these particles (Figure 3) to set a range that has a scattering behavior close to the one searched. Five different samples were tested, and the results plot shows the average of the different samples with their standard deviation. These tests defined the set of concentrations to be used in combination with ink.

From the outcome of these results, 4, 6, and 8 mg/mL concentrations were selected to be tested with the absorbing agent. In addition, 10 mg/mL concentration was added later to achieve the expected values. These changes are detailed in discussions.

The selected concentrations of TiO_2_ were mixed with Indian ink. Samples with different concentrations of each element were tested. Only two different concentrations of ink were used (1 µL/mL and 3 µL/mL), and for each of those concentrations, four different concentrations of TiO_2_ (4 mg/mL, 6 mg/mL, 8 mg/mL, and 10 mg/mL) were mixed. At these different concentrations, it was expected that the desired values of the coefficients ${µ}_{a}\left(\lambda \right)$ and ${µ}_{s}^{\prime }\left(\lambda \right)$ would be obtained.

The differences observed between the films with 1 µL/mL and 3 µL/mL showed that the second group reflected a smaller percentage of light, and the transmission decreased almost to zero in the interval of interest. This outcome was used to calculate the optical coefficients, which increased the ${µ}_{a}\left(\lambda \right)$ values for the set of higher ink concentrations and decreased the scattering of the samples. These results were plotted with reference values obtained from the graphics shown in investigations where the optical coefficients of brain tissues, white matter (WM), and gray matter (GM) were calculated from optical tests.

**Figure 4:**
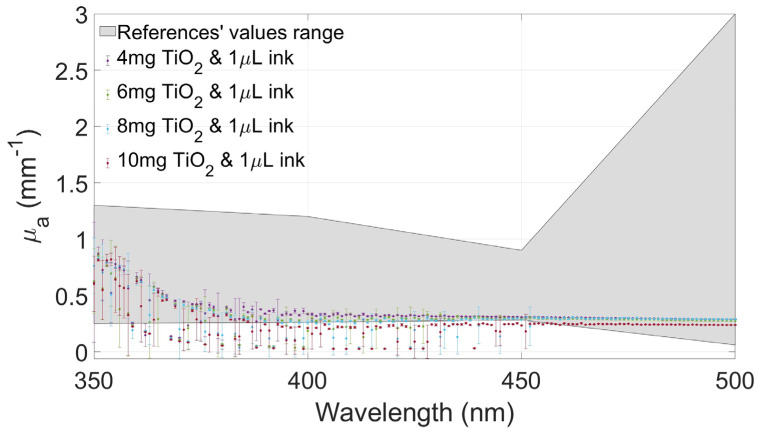
Absorption coefficient values of the films at different concentrations of TiO_2_ with 1 µL/mL of Indian ink and the range values from the literature plotted as a gray area.

**Figure 5:**
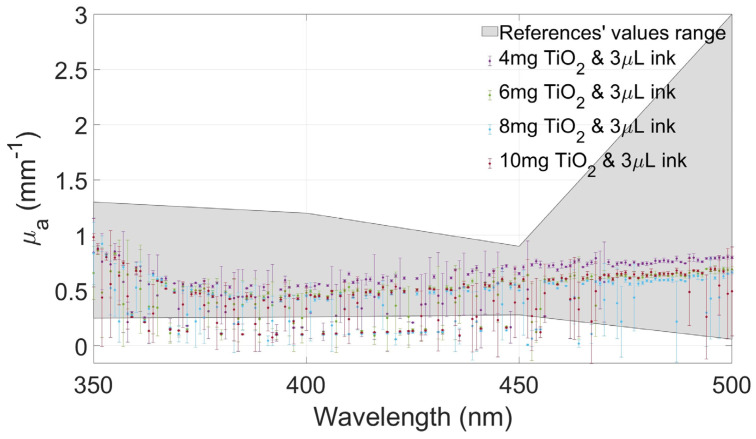
Absorption coefficient values of the films at different concentrations of TiO_2_ with 3 µL/mL of Indian ink and the range values from the literature plotted as a gray area.

**Figure 6:**
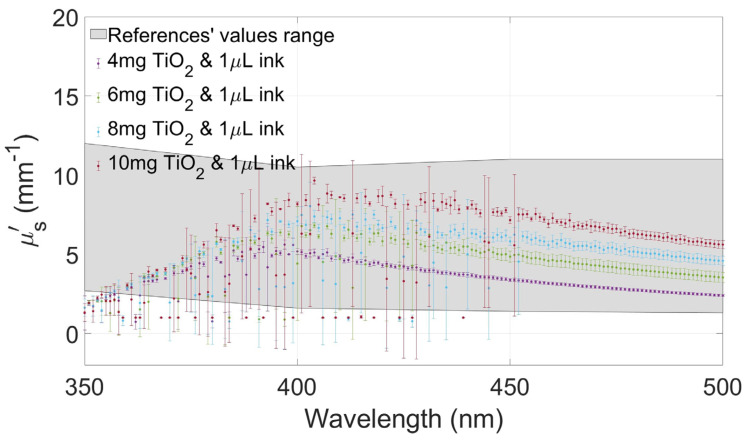
Reduced scattering coefficient values of the films at different concentrations of TiO_2_ with 1 µL/mL of Indian ink and the range values from the literature plotted as a gray area.

**Figure 7:**
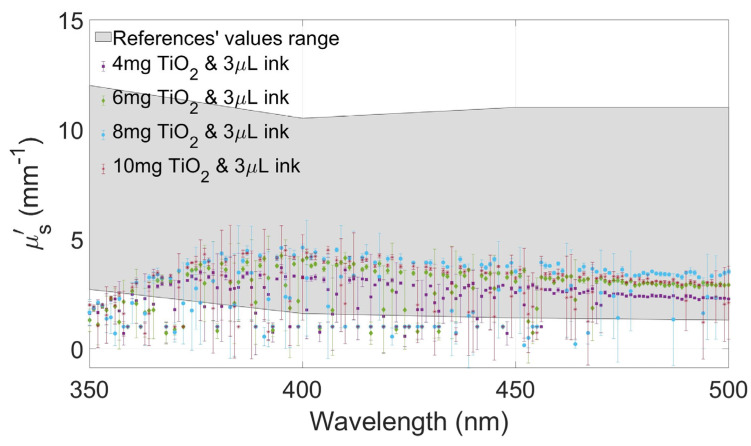
Reduced scattering coefficients of the films at different concentrations of TiO_2_ and 3 µL/mL of Indian ink and the range of values from the literature are plotted as the gray area.

Figure 4, Figure 5, Figure 6 and Figure 7 show the values of ${µ}_{a}\left(\lambda \right)$ and ${µ}_{s}^{\prime }\left(\lambda \right)$ of the developed films and their standard deviation with the four selected concentrations of TiO_2_ with an ink concentration of 1 µL/mL in Figure 4 and Figure 6 and with an ink concentration of 3 µL/mL in Figure 5 and Figure 7. In these graphs, the maximum and minimum values obtained from biological brain tissue [22,23,24,25,26] are plotted as a range, illustrated as the gray area in the figures, for comparison with the values from this study. For plotting these ranges, only data points at 350, 400, 450, and 500 nm wavelengths were used.

As can be observed in the plots of Figure 4 and Figure 5, the differences between the concentrations of Indian ink affect the variation in light absorption, as evidenced by the increase in the absorption coefficient for the concentration of 3 µL/mL (Figure 5) compared to that for the concentration of 1 µL/mL (Figure 4). On the contrary, in the same graphs, it is possible to observe that the effect on absorption due to differences in TiO_2_ concentrations was negligible in our experiments. In other words, although the absorption level has generally increased due to the increase in the amount of Indian ink, the observed absorption coefficients do not show obvious differences with the increase in the TiO_2_ concentration.

Concerning the effect on the variation of the scattering, comparing Figure 6 and Figure 7, it is possible to observe that, in this case, the increase in the variation of TiO_2_ concentration had a direct effect on the reduced scattering coefficients observed in our experiments. Interestingly, the effect is greater with lower light absorption, as seen in Figure 6 concerning Figure 7, where it is observed that a greater absorption of light tends to attenuate the variation of scattering due to the lower amount of transmitted light.

To confirm the effect of the Indian ink and TiO_2_ concentrations on the observable absorption and reduced scattering coefficients of the tissue phantom plots, a series of analyses of variance (MANOVA) were carried out over the values of both coefficients. The MANOVA confirmed that ink is an important factor for changes in the absorption coefficient, with F (2, 1647) = 83.02, *p* < 0.05. The TiO_2_ concentration, with F (6, 3294) = 8.034 and *p* = 0.061, although not strictly statistically significant, was very close to the limit of significance, allowing us to conclude that the variation in TiO_2_ concentration affects the reduced scattering. On the contrary, no main effect was observed for the Indian ink concentration for scattering, while no main effect was observed for the TiO_2_ concentration for absorption. As expected, the absorption coefficient is affected almost only by the absorbing agent used, and the scattering agent mainly defines the reduced scattering coefficient.

**Figure 8:**
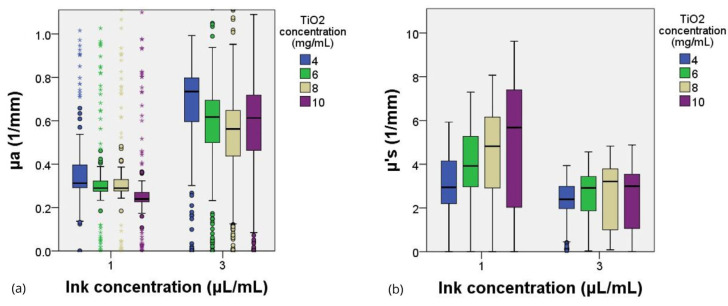
Absorption (**a**) and reduced scattering (**b**) coefficients for different concentrations of TiO_2_, grouped by ink concentration.

Figure 8 shows the differences in the values of reduced scattering and absorption between the different concentrations of TiO_2_ grouped by the ink concentration. It can be seen that the differences in absorption are larger than those in scattering between the two groups of ink concentrations. In addition, it shows how the reduced scattering coefficient values increase as the scattering agent concentration increases.

### 2.4. Optical Coefficients between Phantom and Biological Samples

More detailed information on the observed absorption and scattering values obtained in this work is included in Table 1 and Table 2, respectively, which introduce a summary of reference values obtained in other studies with biological brain tissues. In such tables, the values of ${µ}_{a}\left(\lambda \right)$ and ${µ}_{s}^{\prime }\left(\lambda \right)$ from this investigation are compared to those published in the literature. In addition, only data points at 350, 400, 450, and 500 nm wavelengths were selected for this comparison, and minimum and maximum values for both concentrations of ink are presented to set the range of values that gel films gave as a result.

### 2.5. Discussions

From the results obtained after testing gels with only TiO_2_ (Figure 4), only three concentrations were selected to be combined with the Indian ink. This decision was made based on the reduced scattering values of the different samples. The resulting values from the smaller concentration of the scattering agent (2 mg/mL) were almost within the lower limit of the reference values; therefore, this sample was discarded.

As expected, ${µ}_{s}^{\prime }\left(\lambda \right)$ values decreased when the absorption agent was added to the gels. Therefore, after testing the three selected concentrations of TiO_2_ with Indian ink, another sample with a higher concentration (10 mg/mL) was added for comparison with the others, looking for higher reduced scattering coefficient values within the expected range from the literature.

The values of the absorption and scattering coefficients of real biological brain tissue were found in the literature, even though only a few show values between 350 and 500 nm [22,23,24,25,26]. These values are compared to those calculated from the gels tested in this study, showing that the values of the coefficients changed between the two concentrations of ink. Moreover, the proper combination of both concentrations allows for fabricating gel phantoms with optical coefficients similar to the ones reported in the referenced studies.

When the amount of ink was increased (3 µL/mL), the absorption values increased proportionally, too, and the scattering values decreased, as expected. The scattering property of the gel changed depending on the concentration of both agents. Even with these changes, ${µ}_{a}\left(\lambda \right)$ and ${µ}_{s}^{\prime }\left(\lambda \right)$ are between the values reported from the literature, showing that these combinations of agents are suitable for recreating a brain tissue-mimicking phantom in this range of wavelengths.

The absorption coefficient values of the films with the same amount of ink are very similar, showing a slight difference between the different concentrations of TiO_2_, as shown in Figure 4 and Figure 5. Considerable changes in the absorption are seen when the amount of ink differs, in this case, increases. The absorption values were greater, but the differences between the different films remained similar, regardless of the concentrations of the scattering agent. In other words, this means that the absorption is almost only modified by the ink, regardless of the amount of TiO_2_ in the gel. The prior is supported by the fact that different concentrations exhibited nearly the same behavior.

In the case of the scattering values, the difference between the graphs of the different TiO_2_ concentrations is notorious, but as the amount of ink increased, the plots became similar to each other. The values of the films with higher ink concentrations are close between the different concentrations of TiO_2_, different from those with a smaller amount of ink, in which the graphs are spaced between them. This observation shows that ${µ}_{s}^{\prime }\left(\lambda \right)$ is affected not only by the scattering agent but also by the absorbing agent.

A comparison of the optical properties published in the literature thus far and those obtained in this work is also presented in Table 1 and Table 2. Specifically, the absorption coefficients of the tested gels are closer to the values reported for white brain matter, where the values at 400 nm in this study showed the biggest difference concerning most of the published values. The films with a concentration of 3 µL/mL of ink had greater values of ${µ}_{a}\left(\lambda \right)$ in the tested spectrum, which increased the values at 400 nm but also at 500 nm. These gel values are greater than those reported in the literature. As mentioned before, if the absorption needs to be varied, only the concentration of the absorption agent needs to be modified.

In the case of the scattering coefficient values, the values obtained from the manufactured gels fit better with those reported for gray brain matter in literature. Those gels with an ink concentration of 1 µL/mL obtained reduced scattering values, almost averaged within the range of the published values, compared to the gels with higher ink concentrations, whose values were smaller than most of them. In addition, because of the greater amount of ink, the values between different concentrations of TiO_2_ remained very close to them, causing all of them to not reach the average of the reported range.

The tissue samples used in the reviewed articles were tested from 30 min to 12 h after resection. All of them were stored at low temperatures. The differences between the tests were that Honda et al. [22] used two integrating spheres for the tests, Li et al. [24] used a fiberoptic probe, being the only tests performed without an integrating sphere, and Yaroslavsky et al. [23] obtained their samples from non-diseased human brains during autopsy within 48 h after death. These differences widened the range of values from the consulted references. However, even with these variations, the values obtained from the films tested in this investigation were within this range.

The manufacturing of these phantoms demonstrates that they could be used as brain tissue-mimicking phantoms because their optical property values are within the range of values found in studies conducted directly in brain tissues. This combination of agarose gel mixed with TiO_2_ and Indian ink is suitable for further use in a study focused on the interaction of UV light with brain tissue, which may pave the way for investigations that need the use of brain tissue and may increase the number of studies conducted in the UV range.

## 3. Conclusions

This study proposed a methodology for manufacturing tissue analogs that mimic the optical properties of brain tissue in the UV range using agarose as a phantom matrix, combined with TiO_2_ particles as a scattering agent, and India ink as an absorption agent. Characterization of these samples revealed that the absorption and scattering coefficients obtained fell within the range of values reported for biological brain tissues. This indicates that these agent combinations are suitable for recreating tissue analogs that mimic brain tissue in the UV spectrum.

The results show that the quantity and combination of agents used to manufacture tissue mimics significantly impact their optical properties. Adjusting these variables to achieve the desired similarity with real tissue is crucial. Furthermore, a comparison of the obtained values with literature data demonstrates the validity of the tissue analogs developed in this study. These tissue mimics could have relevant applications in medical research, imaging technique development, and clinical training, providing a versatile and accurate tool for studying and understanding brain tissues in the UV spectrum.

## 4. Materials and Methods

### 4.1. Preparation of Agarose Tissue Simile

Agarose powder (Agarose Basic, IBI Scientific, Dobuque, IA, USA), TiO_2_ particles < 150 nm in size (700347-25G, Sigma-Aldrich, St. Louis, MO, USA), and Indian ink (Drawing ink, Pelikan, Hannover, Germany) were used to fabricate the tissue simile. TiO_2_ particles were used as the scattering agent, and Indian ink was used as the absorption agent. The manufacturing protocol for the samples is as follows.

#### Sample Preparation

1. In a glass container, the quantities $w_{A}$ [g] of agarose, $w_{T}$ [g] of TiO_2_, $v_{i}$ [μL] of Indian ink and v [mL] of distilled water were combined. The combination of the distilled water and Indian ink gives a result of the total volume of water in the mixture, $v_{m}$ [mL]. The amount of agarose will depend on the desired concentration percentage $c_{a}$. The value was determined using Equation (1).

(1) $$c_{a}=\frac{w_{A}}{v_{m}}\times 100,\;$$

The amount of TiO_2_ depends on the desired concentration of the scattering agent in the mixture, as shown in Equation (2).

(2) $$c_{T}=\frac{w_{T}}{v_{m}}\times 100,\;$$

For this study, the amount of Indian ink was set at 1 and 3 µL/mL based on the amount of ink or other carbon-based materials used to fabricate similar phantoms [15,16,26,27,28,29].

2. On a magnetic stirrer, heat the water with agarose, the TiO_2_, and the ink up to melting temperature, approximately 75 °C, while stirring. Once the water is boiling, it is removed from the heat.

The mixture is left to cool down to a temperature of approximately 60 °C before the gel starts to form.

When the desired temperature is achieved, an amount of 8 mL is taken from the preparation and poured into the container, where the gel will cool down to room temperature.

Containers of 52 × 78 mm were designed to obtain gel films of 42 × 40 × 5 mm in volume. They also have a 25.4 × 76.2 mm orifice to perform a transmission test with the created films. They were fabricated in 1.75 mm diameter PLA (polylactic acid) filament material with a density of 1.24 g/cm^3^ and heating the material to 210 °C, using additive manufacturing with a commercial 3D printer (Original Prusa™ i3 MK3S 3D printer, Praga, Czech Republic) with a nozzle diameter of 0.4 mm. The printer was configured to utilize a rectilinear pattern fill with a 20% ratio and a print speed of 200 mm/s, controlled by the PRUSA Slicer software 2.7.0.

A thickness of 5 mm was set considering that the interaction of UV light with the phantoms or even with biological tissue will be helpful only within this depth due to the capacity of light to penetrate the tissue, similar to the type of brain tumor monitoring applications using fluorescence imaging.

### 4.2. Experimental Setup

#### 4.2.1. Objective of the Experiments

The objective of the experiments was to determine the optical coefficients of agarose films with only the scattering agent at different concentrations and with the combination of the scattering and absorbing agent to characterize the fabrication of tissue phantoms with optical properties akin to biological brain tissues in the wavelength range between 350 and 500 nm.

#### 4.2.2. Samples

Films with different concentrations of TiO_2_ were fabricated to determine the differences between them. Four different concentrations were tested (2 mg/mL, 4 mg/mL, 6 mg/mL, and 8 mg/mL) and five films were prepared for each concentration. The total number of films tested was 35.

From the first test, three concentrations of TiO_2_ were selected to be tested in combination with two different concentrations of Indian ink (4 mg/mL, 6 mg/mL, and 8 mg/mL) and another one was added (10 mg/mL). Three films were prepared for the four TiO_2_ concentrations with two sets of Indian ink concentrations. This means that a total of 24 films were tested. The agarose concentration was kept constant for all the manufactured films.

#### 4.2.3. Optical Tests

The transmittance and reflection tests were carried out using the spectrophotometer (Cary 5000 UV-VIS-I, Santa Clara, CA, USA) with an integrating sphere of 110 mm, a sample port diameter of 17 mm, and 0.97 reflectivity of the sphere wall, where gel samples were placed in front of the sphere, so the beam of light passed through the sample inside the sphere for the transmission test, and placed behind it to collect the light reflected, for the reflection tests. These tests were conducted promptly upon preparation of the gels, and when completed, they were discarded. No film was analyzed after the passage of hours or days.

#### 4.2.4. Optical Coefficients Estimation

The data collected from the spectrophotometer represent the percentages of light transmitted and reflected at each wavelength due to the tested samples. The data were normalized to 1 in order to calculate the absorption and reduced scattering coefficients using the method of Inverse Adding-Doubling (IAD).

The IAD method provides a numerical solution to the radiative transport equation with the adding-doubling method, which allows the calculation of transmittance and reflection values by setting absorption and reduced scattering coefficient values. These results are compared to the real values obtained from experimentation to validate if the set values were correct. It can be applied to any medium, but it needs that samples are an infinite plane-parallel slab with a uniform layer and homogenous optical properties [49].

## Figures and Tables

**Table 1:** Values of the absorption coefficient at four different wavelengths reported in published studies and those obtained from the films manufactured in this study.

References	µ_a_ (mm^−1^) @ 350 nm		µ_a_ (mm^−1^) @ 400 nm		µ_a_ (mm^−1^) @ 450 nm		µ_a_ (mm^−1^) @ 500 nm	
	Min. Value	Max. Value	Min. Value	Max. Value	Min. Value	Max. Value	Min. Value	Max. Value
This work’s gels with 1 µL/mL	0.93	1.02	0.208	0.33	0.23	0.31	0.24	0.29
This work’s gels with 3 µL/mL	0.98	1.03	0.45	0.54	0.54	0.66	0.66	0.8
Biotissue White brain matter								
Honda et al. [22]	1		0.9		0.8		0.4	
Li et al. [24]							2.2	
Gebhart et al. [25]			1		0.5		0.2	
Shapey et al. [26]			0.3		0.28		0.22	
Yaroslavsky et al. [23]	0.25		0.3		0.14		0.1	
Biotissue Gray brain matter								
Honda et al. [22]	1.3		1.2		0.85		0.4	
Li et al. [24]							3	
Gebhart et al. [25]			1		0.5		0.25	
Shapey et al. [26]			0.95		0.9		0.38	
Yaroslavsky et al. [23]	0.35		0.26		0.07		0.05	

**Table 2:** Values of the scattering coefficient at four different wavelengths reported in published studies and those obtained from the films manufactured in this study.

References	${µ}_{s}^{\prime }$ (mm^−1^) @ 350 nm		${µ}_{s}^{\prime }$ (mm^−1^) @ 400 nm		${µ}_{s}^{\prime }$ (mm^−1^) @ 450 nm		${µ}_{s}^{\prime }$ (mm^−1^) @ 500 nm	
	Min. Value	Max. Value	Min. Value	Max. Value	Min. Value	Max. Value	Min. Value	Max. Value
This work’s gels with 1 µL/mL	2	2.2	5.17	8.35	3.37	7.47	2.4	5.6
This work’s gels with 3 µL/mL	1.86	2	3.24	4.6	2.7	3.7	2.27	3.5
Biotissue White brain matter								
Honda et al. [22]	10		10		9.7		10	
Li et al. [24]							2.8	
Gebhart et al. [25]			9		8.8		7	
Shapey et al. [26]			10		11		11	
Yaroslavsky et al. [23]	12		10.5		9.24		8.4	
Biotissue Gray brain matter								
Honda et al. [22]	4		4		3.7		3.5	
Li et al. [24]							1.9	
Gebhart et al. [25]			2.8		2		1.9	
Shapey et al. [26]			6		5		4.8	
Yaroslavsky et al. [23]	2.7		1.6		1.4		1.3	

## Data Availability

The data presented in this study are openly available in the article upon request.
